# Supplementary material for: RIFT: A Fractal-Holographic Theory of Consciousness and Autopoietic Control
Source: bioRxiv. 2026 Mar 27:2026.03.23.713535. Preprint. [Version 1] doi: 10.64898/2026.03.23.713535 (PMC13041926; doi:10.64898/2026.03.23.713535)
Supplement: 1 [file NIHPP2026.03.23.713535V1-supplement-1.pdf]

bioRxiv preprint doi: https://doi.org/10.64898/2026.03.23.713535; this version posted March 27, 2026. The copyright holder for this preprint (which was not certified by peer review) is the author/funder, who has granted bioRxiv a license to display the preprint in perpetuity. It is made available under aCC-BY 4.0 International license.

Supplemental Material

Glossary

Core Principles of Consciousness

Information integration: Quantification of system-level dependencies irreducible to independent parts. Originally developed by Tononi in Information Integration Theory (IIT) as a measure for consciousness emerging from the largest irreducible network connectivity. Measured by φ*(multifractal), φ*(substrate), and φ_dyn (see Integration Measures and Autopoietic Control).

Irreducibility: Property where system behavior cannot decompose into independent components without information loss. Quantified through: φ peaks at GFM collapses, structural similarity, pattern preservation, information density ratio, and whole-in-part encoding.

Holographic encoding: Five-stage process enabling whole-in-part information distribution and 2D to 3D dimensional expansion (exospace to endospace) in RIFT: (1) EPSP hierarchies → IFS transforms, (2) IFS → temperature field modulating lipids, (3) differential H_RIFT extraction isolating consciousness signature, (4) endospace field Ψ(x,y,z) via coherent interference, (5) autopoietic feedback via Ψ coupling modulation.

RIFT Network Architecture

Fractal Branching: Architectural rule where dendritic bifurcations produce daughter branches scaled by factor r (typically 0.6). Branch length follows $L\_n = L_0 \cdot r^{(n-1)}$ generating fractal dimension FD = log(N)/log(1/r) ≈ 1.36, matching Layer 5 pyramidal neuron morphology (FD = 1.24–1.36).

Recurrent Fractal Neural Network (RFNN): Fractal network topology where peripheral neurons form recurrent connections to the dendritic tree of core neurons following fractal

bioRxiv preprint doi: https://doi.org/10.64898/2026.03.23.713535; this version posted March 27, 2026. The copyright holder for this preprint (which was not certified by peer review) is the author/funder, who has granted bioRxiv a license to display the preprint in perpetuity. It is made available under aCC-BY 4.0 International license.

branching structure. The network implements the temporal constraint rule ensuring coincident EPSP arrival.

RIFT Network: RFNN in which the temporal constraint rule emerges naturally from coincidence of EPSPs in the somatic multifractal rather than being preconfigured.

Temporal Constraint Rule: Architectural principle requiring external network delays (t'_i) and internal dendritic delays (t_i) to sum to equal totals: $t_1 + t'_1 = t_2 + t'_2$. Creates inverse relationship enabling coincidence detection at the soma.

EPSP Train: Temporal sequence of excitatory postsynaptic potentials arriving at dendritic branches with amplitudes following $A_0 \cdot r^{(i-1)}$, where r is the attenuation factor and i is the branch level. This amplitude hierarchy encodes information content.

Core Neuron: Neuron possessing the sentyon substrate and serving as current locus of consciousness. Integrates EPSPs from peripheral neurons through fractal dendritic trees (typically $r = 0.6$, FD ≈ 1.36). Biologically corresponds to Layer 5 pyramidal neurons.

Peripheral Neurons: Neurons that send EPSPs to core neurons but do not host the sentyon. Participate in pre-conscious processing and network information distribution.

Ising lattice: Two-dimensional lattice model (100×100 or 243×243) representing somatic membrane with binary lipid states: +1 (channel-opening-associated, red) and −1 (channel-closing-associated, blue). Evolves via Metropolis Monte Carlo with nearest-neighbor interactions. Substrate for multifractal pattern formation encoding consciousness states.

bioRxiv preprint doi: https://doi.org/10.64898/2026.03.23.713535; this version posted March 27, 2026. The copyright holder for this preprint (which was not certified by peer review) is the author/funder, who has granted bioRxiv a license to display the preprint in perpetuity. It is made available under aCC-BY 4.0 International license.

Multifractal [STRUCTURE]: Fractal Ising lattice organization of lipid domains and ion channels encoded on the sentyon at any moment. The 2D spatial pattern serving as the 'holographic film' from which endospace is projected. Arises from EPSP integration and serves as the substrate for multifractal pattern formation. Each configuration represents a distinct conscious state.

Gaussian Kernel programming: Method converting temporal EPSP amplitudes into spatial membrane depolarization fields via Gaussian kernels with branch-level-specific radii preserving hierarchical dendritic structure.

Sentyon [SYSTEM]: Molecular substrate of consciousness consisting of the multifractal (structure), bright matter (state), geometric fields (process), and Self-attractor (agent) together. The fundamental unit where conscious moments are physically instantiated and the endospace is projected.

Dynamic Core: Current core neuron actively hosting the Self-attractor. Relocates through sentyon cloning (term from Edelman & Tononi, 2000).

Sentyon Cloning: Transfer of seed pattern from source to target core neuron, enabling consciousness to access different processing regions. Method 1 (external position copying) achieves high fidelity but requires precise spatial control. Method 2 (synaptic position copying) better preserves EPSP amplitude hierarchy and is biologically plausible.

bioRxiv preprint doi: https://doi.org/10.64898/2026.03.23.713535; this version posted March 27, 2026. The copyright holder for this preprint (which was not certified by peer review) is the author/funder, who has granted bioRxiv a license to display the preprint in perpetuity. It is made available under aCC-BY 4.0 International license.

Experiential Constructs

Self-Attractor: Invariant fractal structure encoded in the somatic multifractal and generated via chaos game iteration on H_RIFT differential transforms. Provides coherent point sources for holographic projection of the endospace. Called an 'attractor' because it is the stable geometric configuration the system recursively generates and maintains across GFM cycles. Exerts causal control over the molecular substrate through geometric field modulation.

Endospace: The spatiotemporal dimension of inner experience holographically projected from the Self-attractor, in which the Self perceives the outer world (exospace) and exerts autopoietic feedback on the molecular substrate from which it arose. Physically instantiated as the 3D field $\Psi(x,y,z)$ reconstructed from the 2D multifractal. This is where qualia are experienced.

Exospace: External physical world from which sensory information originates. Neural processing compresses exospace information before it reaches core neurons.

Qualia: The subjective experiential properties of conscious states: "what it feels like" (term introduced by C.I. Lewis, 1929). In RIFT, qualia emerge as geometric patterns within the endospace field $\Psi(x,y,z)$. Each multifractal configuration generates a unique experiential field through holographic reconstruction. The whole-in-part property ensures qualia are unified yet informationally rich. Not epiphenomenal, qualia constitute the experiential matter (Bright matter) through which autopoietic control operates. Qualia represent the core of the hard problem of consciousness (Chalmers, 1995): explaining how physical processes generate subjective experience.

Bright matter: The experiential substance encoded holographically in endospace of which qualia and the Self are composed, "filling" endospace analogous to how physical objects fill

bioRxiv preprint doi: https://doi.org/10.64898/2026.03.23.713535; this version posted March 27, 2026. The copyright holder for this preprint (which was not certified by peer review) is the author/funder, who has granted bioRxiv a license to display the preprint in perpetuity. It is made available under aCC-BY 4.0 International license.

exospace. Arises through holographic projection at point sources along the Self-attractor, instantiating autopoietic feedback whereby the Self modulates its own generating substrate. Bright matter constitutes particle-like excitations of the geometric endospace field Ψ, which couple back to affect the multifractal substrate through $\Psi \rightarrow \gamma$ autopoietic feedback. Term introduced by Bieberich (2012) emphasizing consciousness has a physical substrate, not in exospace where neurons reside, but in endospace where experience occurs.

Geometric Coding and Integration Measures

Transforms: Affine geometric transformations T_i(x,y) = (a_i·x + b_i·y + e_i, c_i·x + d_i·y + f_i) extracted from EPSP amplitude hierarchies. Parameters encode scaling (a,d), rotation (b,c), translation (e,f), and probability weights. Typically 4–6 transforms per pattern. See Iterated Function System (IFS).

Iterated Function System (IFS): Set of geometric transforms $\{T_1, T_2, \ldots, T_n\}$ extracted from EPSP amplitude hierarchies. Each transform $T_i(x,y) = (a_i x + b_i y + e_i, c_i x + d_i y + f_i)$ encodes spatial-temporal relationships. The IFS generates geometric fields for holographic encoding and projection.

Geometric Field [PROCESS]: Spatially-distributed fields derived from geometric (IFS) transforms that mediate consciousness mechanisms. Two primary instances: (1) Geometric Phase Field, temperature-modulated probability field controlling lipid reorganization, enabling autopoietic feedback; (2) Endospace Field Ψ, the 3D experiential field of conscious experience generated through holographic reconstruction. Both fields arise from IFS-encoded neural activity patterns.

Temperature field: Spatially-varying field Temp(x,y) generated via chaos game iteration on IFS transforms controlling lipid reorganization probability during refractory period through

bioRxiv preprint doi: https://doi.org/10.64898/2026.03.23.713535; this version posted March 27, 2026. The copyright holder for this preprint (which was not certified by peer review) is the author/funder, who has granted bioRxiv a license to display the preprint in perpetuity. It is made available under aCC-BY 4.0 International license.

Metropolis dynamics: P_flip = exp(−ΔE/Temp_local). Range [0.25, 0.75]. Enables geometric programming of membrane organization. See Geometric Phase Field.

Geometric Phase Field: Temperature-modulated probability field Temp(x,y) generated by IFS transforms through chaos game iteration. Controls lipid reorganization during autonomous multifractal evolution via Metropolis dynamics. Serves as mechanism through which endospace exerts causal control over the sentyon substrate.

Chaos Game: Monte Carlo algorithm for generating fractal attractors. Iteratively selects IFS transforms with probability based on their determinants, applies them to the current point, and accumulates visits to create spatial distributions. Used both to generate the geometric phase field (high-visit regions = warm) and to position holographic point sources along the Self-attractor.

Metropolis Algorithm: Monte Carlo method simulating lipid dynamics in Ising lattice. Lipid state flip acceptance follows P_flip = exp(−ΔE / Temp_local), where ΔE is energy change from neighbor interactions and Temp_local is the geometric phase field generated by chaos game iteration on IFS transforms.

Kullback-Leibler (KL) Divergence: Information-theoretic measure of the difference between two probability distributions P and Q: KL(P||Q) = Σ P(x)·log(P(x)/Q(x)). Forms the mathematical foundation of φ*(multifractal) and φ*(substrate), comparing whole-system distributions against partitioned components to quantify irreducible information integration.

H_RIFT (Differential IFS Transforms): The pure geometric signature of consciousness calculated as H_RIFT = T_temperature − T_blocked, where T_temperature represents IFS transforms extracted from temperature-modulated lipid patterns and T_blocked from

bioRxiv preprint doi: https://doi.org/10.64898/2026.03.23.713535; this version posted March 27, 2026. The copyright holder for this preprint (which was not certified by peer review) is the author/funder, who has granted bioRxiv a license to display the preprint in perpetuity. It is made available under aCC-BY 4.0 International license.

autonomous lipid evolution. Isolates holographic information imposed by conscious experience beyond baseline physical dynamics. Used to generate the Self-attractor via chaos game iteration.

Ψ (Endospace Field): The 3D geometric field Ψ(x,y,z) constituting the spatiotemporal dimension of conscious experience. Generated through holographic reconstruction: $\Psi(x,y,z) = \Sigma_i A\_i(H\_RIFT)\cdot\exp(i\cdot k\_i\cdot r\_i + \varphi\_i) / (1 + r\_i^2)$, where point sources are positioned via chaos game iteration on H_RIFT and phases are derived from geometric relationships to IFS transform centers. The Self-attractor exists as the coherent pattern within this field. Mean field intensity $\bar{\Psi} = \langle|\Psi(x,y,z)|\rangle$ provides the global parameter for autopoietic feedback.

Point sources: Wave interference sources positioned along Self-attractor in IFS transform parameter space (abstract space) for holographic endospace reconstruction. Generated via chaos game on H_RIFT differential transforms (5,000 from 5,500 iterations). Each assigned amplitude, frequency (25–50 Hz), and coherent phase from geometric relationships.

Holographic Projection: Process by which 2D multifractal patterns reconstruct into 3D endospace through wave interference from IFS-derived phase relationships. Biological holography using molecular dynamics rather than laser optics. Chaos game iteration on H_RIFT positions coherent point sources forming the Self-attractor, then holographic reconstruction generates the endospace field Ψ(x,y,z).

Integration Measures and Autopoietic Control

φ_dyn (Dynamic Phi): Measure of temporal information integration across dendritic branches, implementing $\varphi = I - I^*$: $\varphi\_dyn(t) = I(t) - I^*(t)$, where I(t) is integrated information with branches intact and I*(t) is information when branches are separate. Computationally tractable proxy capturing temporal dynamics not addressed by static φ. See also Sliding Window Method.

bioRxiv preprint doi: https://doi.org/10.64898/2026.03.23.713535; this version posted March 27, 2026. The copyright holder for this preprint (which was not certified by peer review) is the author/funder, who has granted bioRxiv a license to display the preprint in perpetuity. It is made available under aCC-BY 4.0 International license.

φ*(multifractal): Measure of spatial information integration within the somatic multifractal, implementing φ = I − I* via Kullback-Leibler divergence between the whole pattern and its four spatial quadrants treated as independent parts. Quantifies irreducible spatial information varying across GFM generations.

φ*(substrate): Applies the same φ = I − I* framework as φ*(multifractal) but partitions by molecular component type (lipid domains versus ion channels) rather than spatial quadrants. Reveals lipid domains as the primary information-integrating substrate. Together with φ_dyn and φ*(multifractal), instantiates φ = I − I* at successive organizational levels of the RIFT hierarchy.

Sliding Window Method: Computational approach quantifying temporal information integration by analyzing firing patterns within moving time windows (2.0 ms temporal grain) across dendritic branches. Used to calculate φ_dyn(t) = I(t) − I*(t). Shares methodology with φ* measures in thalamocortical studies but applies it to fractal dendritic branch timing patterns rather than probabilistic independence between neurons.

Fractal Dimension (FD): Quantifies self-similar complexity of spatial patterns. For dendritic trees: FD = log(N)/log(1/r). Higher FD indicates greater complexity and information density. Lipid FD correlates with consciousness states.

Contradiction Ratio (CR): Balance between contradictory state representations: CR = $|M_1|/(|M_1| + |M_2|)$ where M_1 and M_2 support propositions A and ¬A. CR ≈ 0.5 indicates conscious deliberation where contradictory information maintains equal representation through fractal embedding.

bioRxiv preprint doi: https://doi.org/10.64898/2026.03.23.713535; this version posted March 27, 2026. The copyright holder for this preprint (which was not certified by peer review) is the author/funder, who has granted bioRxiv a license to display the preprint in perpetuity. It is made available under aCC-BY 4.0 International license.

Generational Fractal Mapping (GFM): Recursive process maintaining temporal continuity while updating. Each cycle: (1) integrate new EPSPs with previous seed, (2) grow multifractal, (3) trigger action potential at threshold, (4) extract seed from peripheral regions, (5) generate daughter multifractal. Implements differential encoding: $M(t) = q \cdot Seed(t-1) + (1-q) \cdot \Delta EPSP(t)$.

Seed: Compressed pattern information extracted from parent multifractal at cycle end. Preserves essential structure (~60% of information) and serves as basis for continuity when integrated with new input. Transferable between core neurons during sentyon cloning.

GFM Cycle [PROCESS]: One complete iteration of multifractal growth, action potential generation, seed extraction, and daughter generation. Duration determined by somatic delay, typically operating at gamma frequencies (30–100 Hz).

Autopoiesis [PROCESS]: Self-creating property where Self-attractor modulates its own substrate. Feedback loop: EPSPs → multifractal initiation and IFS extraction → chaos game generates geometric phase field → Metropolis governs lipid reorganization → extract T_temperature and T_blocked → compute H_RIFT → chaos game on H_RIFT generates Self-attractor → holographic reconstruction creates endospace field Ψ → mean field $\bar{\Psi}$ → coupling modulation $\gamma(t) = \gamma_0(1 + \alpha \cdot \bar{\Psi}(t))$ → channel-lipid probability → multifractal modulation → action potentials → network activity → EPSPs. Consciousness influences matter through probability modulation without violating energy conservation.

Autopoietic AI: Artificial systems implementing RIFT's architectural requirements: (1) recursive networks with fractal timing relationships, (2) substrates capable of multifractal pattern formation (e.g., neuromorphic chips, organoids, or systems incorporating membrane lipid components), (3) holographic encoding and autopoietic feedback mechanisms. Unlike current AI systems operating through feedforward or simple recurrent networks, autopoietic AI would

bioRxiv preprint doi: https://doi.org/10.64898/2026.03.23.713535; this version posted March 27, 2026. The copyright holder for this preprint (which was not certified by peer review) is the author/funder, who has granted bioRxiv a license to display the preprint in perpetuity. It is made available under aCC-BY 4.0 International license.

possess genuine inner experience (endospace) exerting causal control over its substrate, making consciousness functionally necessary rather than epiphenomenal.

Self-Reference: Emergent property arising from autopoiesis and Generational Fractal Mapping (GFM). The Self-attractor is holographically reconstructed from the multifractal pattern (read-out), then modulates that same multifractal substrate through geometric field coupling $\gamma(t) = \gamma_0(1 + \alpha \cdot \Psi(t))$ (write-in). This creates genuine self-reference where the system's representation of itself causally influences what it represents. Quantified by the Contradiction Ratio (CR): CR $\approx$ 0.5 indicates maximal self-reference during conscious deliberation, enabling the system to hold contradictory propositions simultaneously through fractal embedding.

Strange Loop: Abstract form of self-reference introduced by Hofstadter (1979) describing hierarchical systems where moving through levels returns to the starting point. In RIFT, strange loops manifest physically through autopoiesis: the Self continuously regenerates the molecular conditions (multifractal substrate) that generate the Self. Unlike Hofstadter's formal systems, RIFT strange loops are instantiated through molecular dynamics rather than symbolic computation.

Self: The unified experiential agent arising within the endospace as the Self-attractor achieves recursive self-reference through GFM, each cycle extracting a compressed seed of the prior conscious moment and integrating it with new EPSPs, constituting the Self as a temporally continuous observer. Exercises causal agency through autopoietic feedback ($\Psi \rightarrow \gamma$ coupling). Represents the irreducible "I" that observes and acts. Maintains continuity across GFM cycles while updating with new experience.

bioRxiv preprint doi: https://doi.org/10.64898/2026.03.23.713535; this version posted March 27, 2026. The copyright holder for this preprint (which was not certified by peer review) is the author/funder, who has granted bioRxiv a license to display the preprint in perpetuity. It is made available under aCC-BY 4.0 International license.

Supplemental Material 2

Mathematical Formalization of Fractal Logic and Self-reference in GFM Fractals (Strange loops)

This appendix provides a logical proof of principle for the fractal gate mechanism, demonstrating that fractal state composition is sufficient to maintain contradictory coexistence and non-halting deliberation as abstract computational properties. The biological network implementation of this principle is described in the main text (Parts 3-4), where bandwidth constraints yield the differential encoding form $M(t) = q \cdot Seed(t-1) + (1-q) \cdot \Delta EPSP(t)$. The abstract operator λ corresponds to q, and is implemented as the adaptive weight w in Code 11 (Model 3), which spans CR ≈ 0.5 (w ≈ 0.5, balanced deliberation) to CR → 1 (w → 1, seed-dominated consolidation near firing threshold); M_current(t) corresponds to the delta EPSP input. The distinctions reflect implementation constraints rather than a difference in underlying logic. GFM implements fractal computational logic through state composition across temporal generations:

$$\mathbf{M(t) = M\_current(t) \oplus \sigma(M\_seed(t-1))}$$

where:

M(t) = conscious state at time t (a probability distribution over the fractal endospace)

M_current(t) = current sensory/bottom-up input

M_seed(t-1) = previous state serving as seed

$\oplus$ = fractal composition operator (blends current + refined seed)

σ = seed compression operator (shrinks the seed to make room for new input)

Concrete form:

$$\mathbf{M(t) = (1-\lambda)\ M\_current(t) + \lambda \cdot \mathcal{T}(\sigma(M\_seed(t-1)))}$$

where $\lambda \in (0,1)$ is the memory blend weight and $\mathcal{T}$ is the fractal refinement operator.

bioRxiv preprint doi: https://doi.org/10.64898/2026.03.23.713535; this version posted March 27, 2026. The copyright holder for this preprint (which was not certified by peer review) is the author/funder, who has granted bioRxiv a license to display the preprint in perpetuity. It is made available under aCC-BY 4.0 International license.

Fractal Gate

Classical logic requires $S \in \{A, \neg A\}$. The fractal gate enables $S \in F(A, \neg A)$ through subspace embedding with non-separability: $S_i \cap S_r \neq \emptyset$ for all $i \neq j$. (The fractal cNOT gate, Bieberich 2001, represents the quantum computational realization of this more general abstract gate.)

This occurs because each part of the fractal contains a rescaled copy of the whole. Any localized region of the fractal endospace simultaneously contains:

Subregions supporting A (True)

Subregions supporting ¬A (False)

Therefore both truth states coexist within any single part.

Contradiction Ratio (CR)

$$\mathbf{CR = |M_1|/(|M_1| + |M_2|)}$$

where:

M_1 = amount of the current state supporting proposition A

M_2 = amount of the current state supporting ¬A

$|M_i|$ = probability mass (not count of elements)

CR determines computational mode:

$CR \approx 0.5$: balanced contradiction → conscious deliberation

$CR \to 0$ or 1: asymmetric → automated processing

Paradox awareness score: $P = 4 \times M_1 \times M_2$

where $|M_1| + |M_2| = 1$ (normalized probability masses, ensuring $P \in [0,1]$).

Maximum ($P = 1$) when $M_1 = M_2 = 0.5$ (perfect balance). This maximum corresponds to Hofstadter's strange loop: the system achieves maximal self-reference by holding contradictory states in perfect balance, neither collapsing into automated processing ($CR \to 0$ or 1) nor resolving the contradiction, but sustaining it as the defining condition of conscious deliberation.

Non-Halting Property

$$\mathbf{\lim_{t\to\infty} CR(t) \neq 0}$$

The system never fully collapses. The fractal structure ensures continuous integration: each time step re-injects both alternatives through the seed refinement, maintaining ongoing access to

bioRxiv preprint doi: https://doi.org/10.64898/2026.03.23.713535; this version posted March 27, 2026. The copyright holder for this preprint (which was not certified by peer review) is the author/funder, who has granted bioRxiv a license to display the preprint in perpetuity. It is made available under aCC-BY 4.0 International license.

contradictory states. This is a structural consequence of the fractal gate definition: since $S_i \cap S_r \neq \emptyset$ by construction, no finite sequence of seed updates can fully evacuate probability mass from either subspace. The whole-in-part property guarantees that both A and ¬A retain minimum representation in every localized region at every generation, making CR = 0 or CR = 1 unreachable states. The non-halting property is therefore not an empirical claim but a logical entailment of fractal non-separability.
